# Takotsubo cardiomyopathy: ten year experience at a tertiary care hospital in Pakistan

**DOI:** 10.1186/1756-0500-7-932

**Published:** 2014-12-19

**Authors:** Abid Hussain Laghari, Aamir Hameed Khan, Khawar Abbas Kazmi

**Affiliations:** Senior Instructor, Cardiology section, Department of Medicine, Aga Khan University Hospital, Karachi, Pakistan

**Keywords:** Tako-tsubo Cardiomyopathy (TTC), Stress induced cardiomyopthy, Left ventricular systolic dysfunction, Right ventricular dysfunction

## Abstract

**Objective:**

There is very little literature regarding Takotsubo Cardiomyopathy (TTC) from the Asian Countries other than Japan and Korea. We conducted this study to determine the demographics, clinical presentations, complications and recovery of left ventricular (LV) systolic function in TTC patients of Pakistani origin.

**Methods:**

A ten years retrospective case series study of TTC was conducted at the Aga Khan University Hospital. Patients were followed for up to six months after presentation, with special emphasis on the recovery of LV function.

**Conclusion:**

TTC is classically triggered by an acute illness or by extreme stress and a triggering incident may not always be identified. It usually presents in the guise of an acute coronary syndrome (ACS). Our data was congruent with the existing literature, except for more heart failure and cardiogenic shock. Average Troponin-I (Tn-I) levels were also higher as compared to western population. The reason for more severity in our patients may be late presentation or different level of response to stress.

## Background

Tako-tsubo cardiomyopathy (TTC) also called stress-induced cardiomyopathy, apical ballooning syndrome or broken heart syndrome. TTC is usually characterized by temporary systolic dysfunction of left ventricular apex and/or mid segments that mimics myocardial infarction (MI), but in the absence of obstructive coronary artery disease [1–7]. TTC was 1^st^ defined in Japan and was afterwards reported in non-Asian populations, as well as the United States and Europe [1, 8]. In the usual type of cardiomyopathy, the contractile function of the mid and apical segments of the left ventricle (LV) are reduced, and there is compensatory hyperkinesis of the basal segments, creating a balloon-like appearance of the distal LV with systole. Less commonly (17%), the ventricular hypokinesis is restricted to the mid-ventricle with relative sparing of the apex [9]. An association with acute illness, extreme stress, myocardial bridging of LAD has been reported in literature [1, 2, 5, 10]. There are few case reports on TTC from our part of the world but no large studies. Since TTC is a rare disorder, so numbers of patients included have been small. We therefore conducted this study at a tertiary care university hospital spanning over a ten years period.

**Figure 1:**
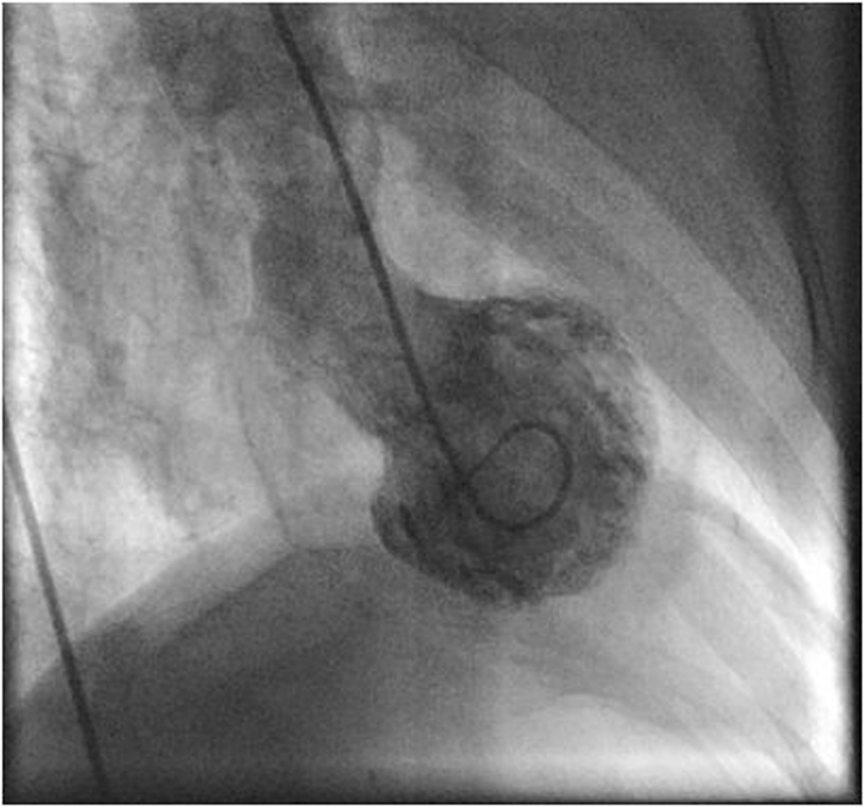
**Left Ventriculogram RAO View: Showing typical apical ballooning and hypercontractile basal segments.**

## Methods

A descriptive case series study done at the Aga Khan University Hospital. The patient’s medical files were reviewed from 1^st^ July 2002 to 30^th^ June 2012. Formal exemption from the University ethics review committee was obtained before embarking on the study. Files of patients diagnosed with TTC were reviewed and information for age, gender, co-morbid condition, any associated stress, clinical presentation, ECG changes, coronary angiogram or ventriculography findings, cardiac complications during hospital stay and number of days of hospital stay were retrieved on a preplanned pro forma. Subsequently, transthoracic echocardiograms, which were done between one and four weeks of presentation were reviewed to see improvement in left ventricular systolic functions.

### Diagnostic criteria for TTC

There are multiple diagnostic criteria for TTC and there is lack of consensus among the experts on single diagnostic criteria (Ethics review committee). We have applied the commonly used modified Mayo clinic diagnostic criteria for TTC. The following four features were necessary for the diagnosis of TTC; (1) temporary hypokinesis, akinesis or dyskinesis of the LV mid segments with or without apical contribution. The regional wall motion abnormalities that classically extend beyond a single coronary artery supply; (2) lack of obstructive coronary artery disease (CAD) or angiographic proof of acute plaque rupture; (3) New ECG changes (either ST-segment elevations and/or T wave inversions) or modest rise in cardiac enzyme troponin-I; (4) No identifiable pheochromocytoma or myocarditis ( Clinically and on follow up).

The data was cross checked by reviewing the files and only those patients were included in the study who met the above mentioned diagnostic criteria.

### Recovery of LV function

This was defined as echocardiographic documentation of improvement in left ventricular ejection fraction (LVEF) > 50%.

## Results

Twenty nine patients were identified through the hospital data base with primary and secondary diagnosis of TTC. Twenty six (89.65%) patients were female and three (10.34%) were male. Patient’s age range was 31 to 86 years and mean age was 61.2 years. Fourteen (48.27%) patients were hypertensive and three (10.34%) had diabetes mellitus. Twenty one (72.41%) patients presented with chest pain and all patients were in heart failure. Nineteen patients (65.51%) had cardiogenic shock. In our study ECG findings of ST elevation MI were present in 19 (65.51%) patients. ECG changes were; anterior ST elevations in twelve (41.39%), inferolateral ST elevation in four (13.79%), lateral ST elevations in three (10.34%) patients. ST depression in inferolateral leads in two (6.89%) patients. Deep T wave inversions in precordial leads (wellens’ type) were present in three (10.34%), prolonged QT intervals in two patients and nonspecific ST changes in two patients. Troponin I (Tn-I) were raised in all patients. Stress factors were noted in eighteen (62.06%) patients, which included; demise of a dear one in three patients, post-surgical procedure in nine patients and recurrent vomiting in six patients. Vomiting was due to urinary tract infection (UTI) and fever in three patients, cholelithiasis in one patient and secondary to anti-tubercular treatment (ATT) in two patients. The left ventricular ejection fraction (EF) at presentation was 10 to 15% in seven (24.13%) patients, 20 to 25% in six (20.68%), 30 to 35% in thirteen (44.82%) patients, 40 to 45% in three (10.34%) patients. On coronary angiogram, coronary arteries were normal in twenty (68.96%) patients and non-obstructive in nine (31.03%) patients. Majority of patients (89.65%) had apical variant of TTC and three patients had mid left ventricular variant. Intra-aortic balloon pump (IABP) support was needed in seventeen (58.62%) patients and fourteen (48.27%) patients required mechanical ventilation. Repeat echocardiogram between 1 to 4 weeks showed normal left ventricular systolic functions in all patients. Mean hospital stay was 8.5 days and all patients were discharged from hospital in stable condition. Patients were followed through clinic visit records; two patients had recurrent TTC and both were female patients.

## Discussion

### Presentation and patient bio data

Over ten years twenty nine cases were identified with mean age of 61.2 years. Most of patients were women and only three were men. In published studies TTC has been reported much more frequently in females than males and females accounted for 80 to 100% of cases [7, 8]. In the review of other case series a mean age of 61 - 76 years was reported [7]. TTC is suspected in patients who present with an acute coronary syndrome after extreme stress in whom the clinical manifestations and ECG changes are out of proportion to the degree of rise in cardiac enzymes [3]. The patients of TTC present in the same way as that of ACS [1, 11, 12]. The acute retrosternal chest pain is the most common presenting symptom. In our study significant number of patients (72.41%) had cardiac chest pain on presentation and all patients were in heart failure. In other case series, TTC accounted for 1.7 - 2.2% of cases presenting with suspected ACS [13–17].

### ECG findings

Significant ECG Changes were present in majority of patients and ST segment elevations were the predominant ECG findings. In a systematic review of 14 series, the main findings were; ST elevations were present in 34 - 56% of patients [14, 18, 19]. In reported literature, ST segment elevations are most commonly noted in the anterior chest leads. Deep T wave inversion with QT interval prolongation, abnormal Q waves, non-specific abnormalities have also been reported [12, 13]. The Wellens’ type electrocardiogram (ECG) pattern is suggestive of myocardial edema and is observed in clinical conditions characterized by reversible left ventricular (LV) dysfunction, either ischemic or nonischemic [20].

### Cardiac enzymes

As per ACS protocol, Tn-I was checked at time arrival in emergency room and second Tn-I was checked after eight hours of the first sample. The rise in cardiac biomarkers was mild, which in contrast with a severe hemodynamic compromise. In the reported systematic review the cardiac enzyme levels were elevated in 86% of patients and troponin T levels ranged from 0.01 - 5.2 ng/mL [14, 21]. In our study Tn-I levels range from 0.56 - 25.4 ng/ml and a mean of 10.80 ng/ml, which were higher compared to other series.

### History of stress factors or illness

In our study the history of stress factors was present in eighteen (62.06%) patients. The remaining eleven (37.93%) patients had no history of apparent stress. In the reported studies the onset of TTC is frequently associated with acute illness or extreme physical or emotional stress [1].

### Echocardiographic findings

The echocardiography or left ventriculography typically demonstrates the classical apical ballooning [1, 2, 14]. In other studies an average left ventricular ejection fractions (EF) of 20 to 49% has been reported [1, 2, 12, 14] (Figure 1). In our study the left ventricular ejection fraction was severely reduced (LV EF < 30) in about half of patients. Two patients had left ventricular apical thrombus. In literature LV apical thrombus formation and cardioembolic stroke have also been reported [21].

### Coronary angiogram findings

In our study all patients underwent coronary angiogram and showed normal coronary arteries in nineteen (65.51%) patients and non-obstructive coronary arteries in ten (34.48%) patients. Five (17.24%) patients had plaque in LAD. A possible role for atherosclerotic plaque rupture and thrombosis with spontaneous recanalization has not been well proposed and the outcomes of intravascular ultrasound (IVUS) studies are mixed [22–24].

### Type of TTC

In our study majority of patients had the apical variant. The clinical presentation of typical and apical sparing variants appear to be similar [3, 25]. In a reported case series study, 82% of case were apical, 17% mid-ventricular, and 1% basal [9]. In our study only three (10.34%) patients had the mid LV variant.

### Mechanical ventilation and IABP

In patients with significant LV dysfunction associated with cardiogenic shock Intra-aortic balloon pump (IABP) is the preferred therapy [1]. We had nineteen (65.51%) patients in cardiogenic shock, in whom seventeen (58.62%) patients needed intra-aortic balloon pump (IABP) support. In a review of 12 prospective series, pulmonary edema occurred in 0 to 44% of cases and IABP was used in 0 - 18% of patients [7]. In our study fourteen (48.27%) patients required mechanical ventilator due heart failure and pulmonary edema. Why did more patients require IABP and mechanical ventilation in our study? This is more likely to be possible late presentation and lack of initial good first aid. The other reason may have been poorly controlled other risk factors like hypertension and diabetes. Ethnicity may also play a role, which can remain speculative at best.

### In hospital outcome

The reported in-hospital mortality rates range from 0 – 8% [1, 7, 11, 12]. Despite the severity of the acute attack, TTC is a temporary disorder treated with supportive therapy. Patients who survive the acute event classically recover their ventricular function to normal within 1 to 4 weeks [1, 2, 12]. In our study there was no in hospital mortality.

### Recovery of left ventricular functions

In our study repeat echocardiogram between 1 to 4 weeks showed normal left ventricular systolic functions in all patients. Mean hospital stay was 8.5 days and all patients were discharged from hospital in stable condition.

### Limitations of the study

This is a retrospective study done through review of patient’s records. The possibility of missing few patients due to file coding remains a clear limitation.

## Conclusion

TTC is classically triggered by an acute illness or by extreme stress and a triggering incident may not always be identified. ACS like presentation is the most common. Our data was congruent with international studies except higher cardiac biomarkers, more heart failure and cardiogenic shock. Average Tn-I levels were also modestly high as compared to western population. There was no in hospital mortality and two cases had recurrence.
